# *trans*-Bis[2-(pyrimidin-2-yl)ethyn­yl]bis­(tri­phenylphosphine)palladium

**DOI:** 10.1107/S2414314625006364

**Published:** 2025-08-05

**Authors:** Eric Bosch

**Affiliations:** a Chemistry and Biochemistry Department, Missouri State University, 901 South National Avenue, Springfield MO 65897, USA; Katholieke Universiteit Leuven, Belgium

**Keywords:** crystal structure, Sonogashira coupling reaction, bis alkynylpalladium complex

## Abstract

The title palladium complex, [Pd(C_6_H_3_N_2_)_2_(C_18_H_15_P)_2_], was isolated as an impurity that co-eluted with the organic product of a palladium-catalyzed Sonogashira coupling. It crystallizes in the monoclinic space group *C*2/*c* with one half of the complex in the asymmetric unit. The palladium atom (site symmetry ) has a square-planar geometry with *trans* phosphine ligands. The P—Pd—C angle is 95.19 (9)°.

## Abstract 2

The the title compound, isolated after a Sonogashira coupling reaction, crystallizes in the monoclinic space group *C*2/*c*.

## Graphical abstract

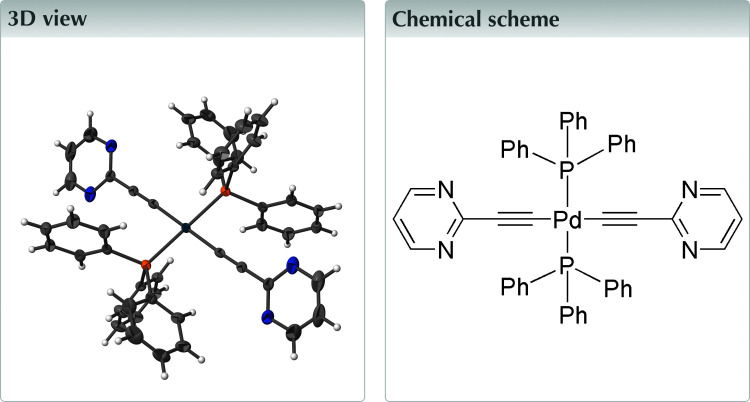

## Structure description

The Sonogashira reaction is a palladium-catalyzed cross-coupling reaction of aryl- and vinyl­halides with terminal alkynes to form alkynyl­benzenes (Sonogashira *et al.*, 1975 ▸). The mechanism involves a transient inter­mediate in which the aryl and alkynyl moieties are both bound to the palladium atom. Subsequently, reductive elimination yields the aryl alkyne. The intentional formation of mono- and bis-aryl­ethynyl palladium complexes has separately been exploited in a variety of fields, including self-assembled metallocycles (Sepehrpour *et al.*, 2019 ▸) and nonlinear optical materials (Yang *et al.*, 1995 ▸). Herein we describe the structure of the title bis-alkynyl palladium complex, [Pd(C_6_H_3_N_2_)_2_(C_18_H_15_P)_2_], fortuitously isolated during the workup of a Sonogashira coupling reaction performed with a slight excess of 2-ethynyl­pyrimidine.

**Figure 1:**
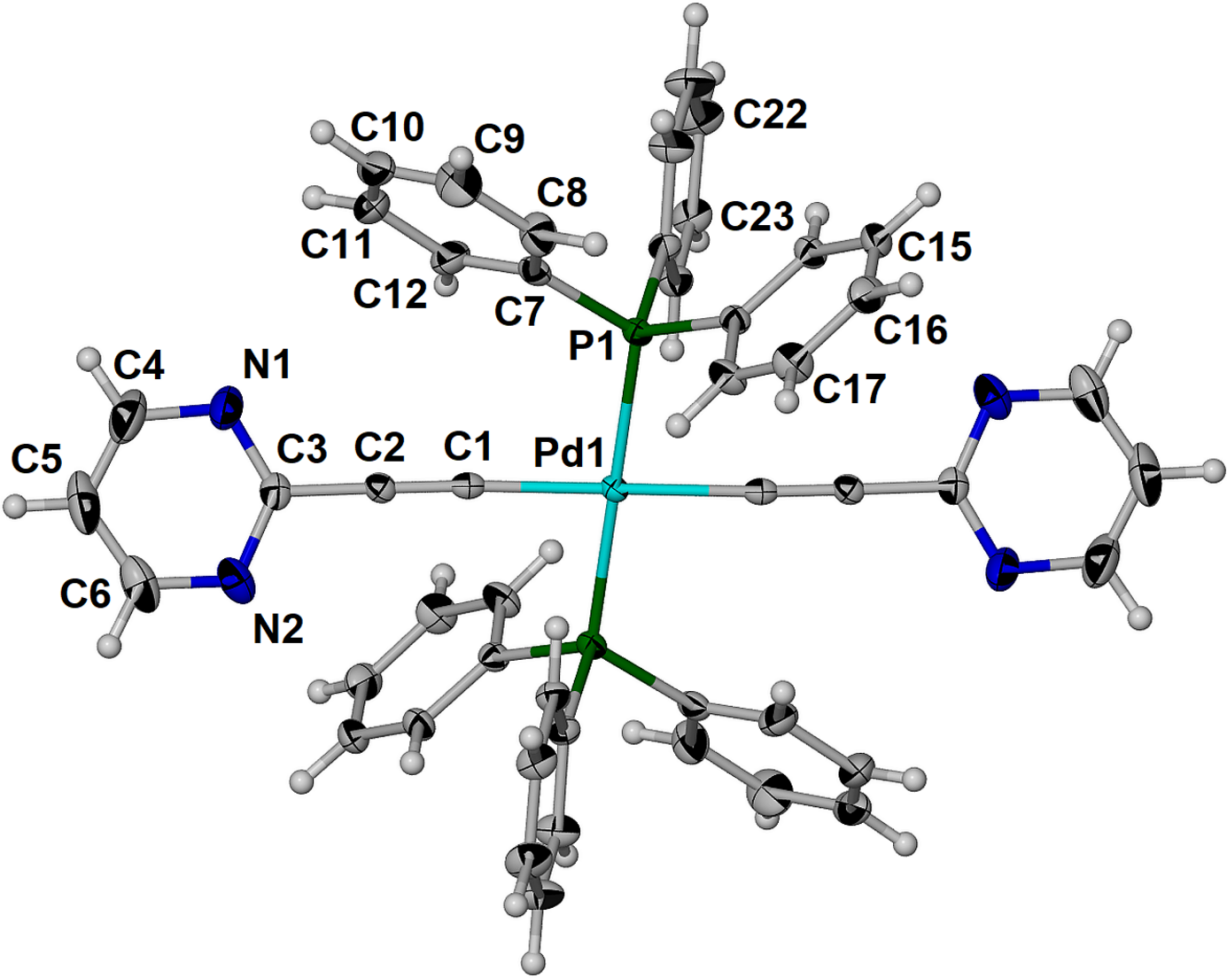
Partially labeled asymmetric unit of the the title palladium complex, [Pd(C_6_H_3_N_2_)_2_(C_18_H_15_P)_2_], including the symmetry-generated half of the complex (symmetry operation: 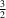 − *x*, 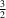 − *y*, 1 − *z*).

The asymmetric unit contains one half of the complex as shown in Fig. 1 ▸. The palladium atom adopts square-planar geometry with symmetry-constrained linear P—Pd—P and C—Pd—C bonds and a P1—Pd1—C1 angle of 95.19 (9)°. The pyrimidine rings are twisted by 28.42 (9)° relative to the plane defined by the four atoms directly bonded to palladium. The Pd1—C1 distance is 2.008 (3) Å. A search of the Cambridge Structural Database (CSD, version 2025.1.1; Groom *et al.*, 2016 ▸) for related bis-ethynyl bis-phosphino palladium complexes yielded 14 unique structures. In these structures, the Pd—C distance ranges from 1.986 to 2.069 Å with an average of 2.012 Å.

**Figure 2:**
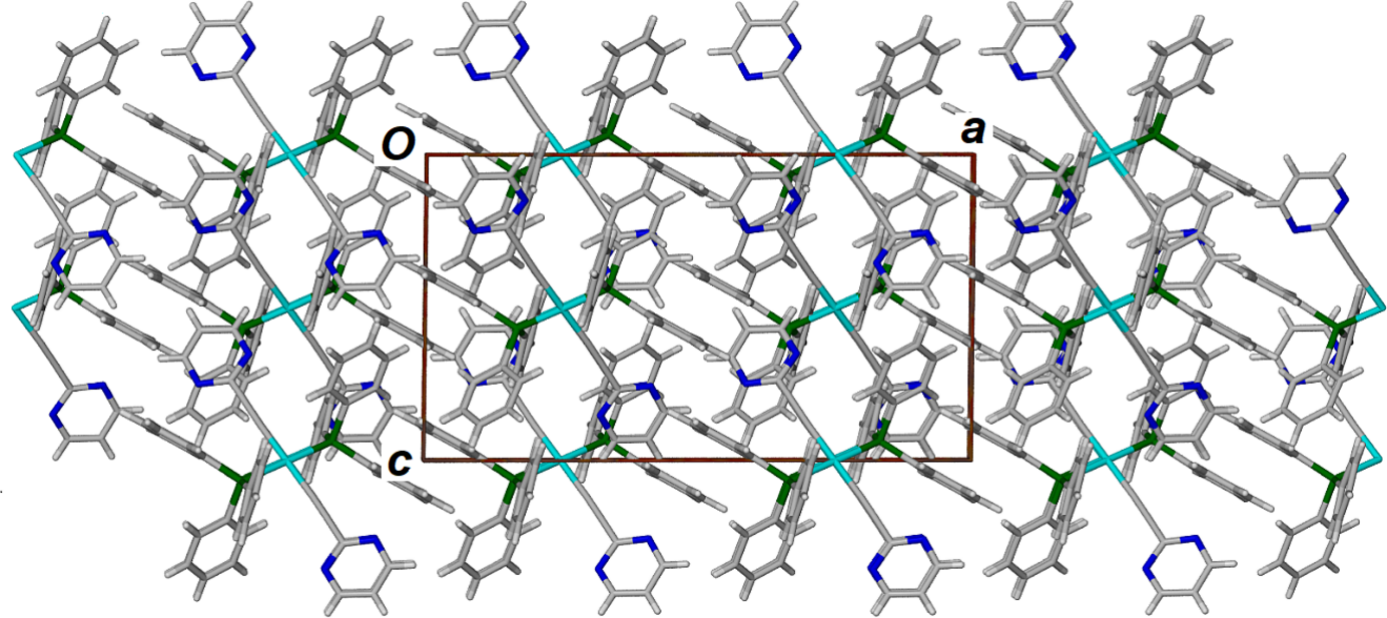
View along the *b* axis of the crystal packing of the title palladium complex [Pd(C_6_H_3_N_2_)_2_(C_18_H_15_P)_2_].

**Figure 3:**
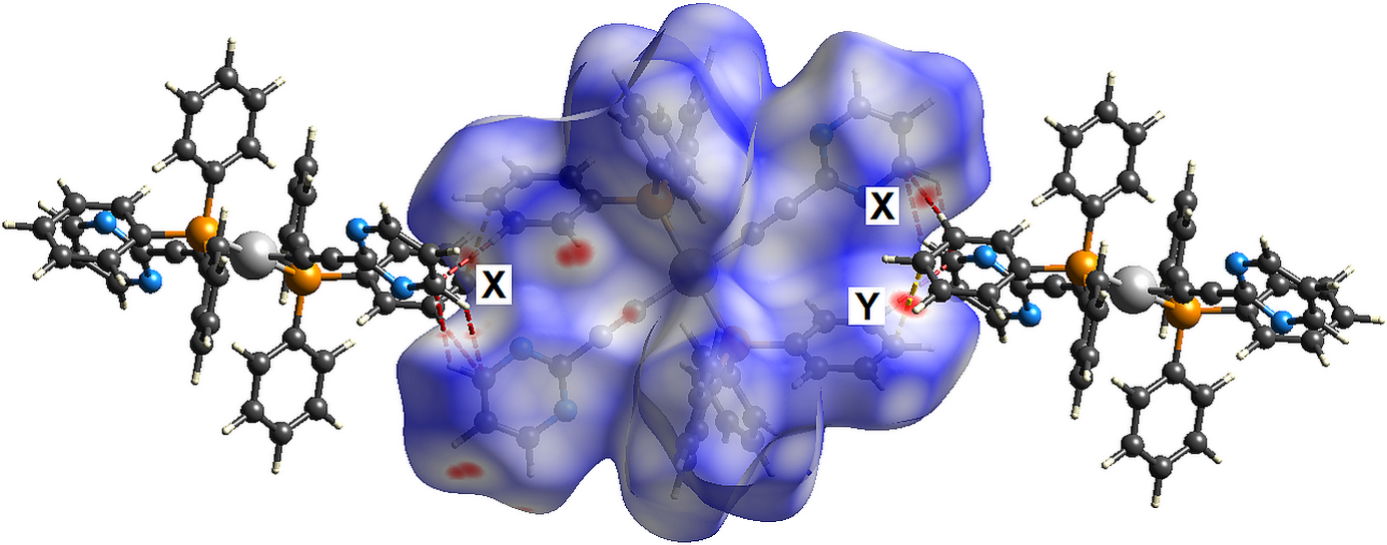
Hirshfeld surface of the title palladium complex [Pd(C_6_H_3_N_2_)_2_(C_18_H_15_P)_2_], with two adjacent inter­acting complexes. C⋯H and C⋯C inter­molecular inter­actions are shown as red and yellow dashed lines, respectively, and labelled X and Y.

The crystal packing (Fig. 2 ▸) along with a view of the Hirshfeld surface (Fig. 3 ▸; Spackman *et al.*, 2021 ▸) show that, as expected, the major inter-complex inter­actions involve inter­digitation of the aromatic moieties. Accordingly, element-to-element analysis of these inter­actions shows that major inter­actions are H⋯H, C⋯H/H⋯C and N⋯H/H⋯N inter­actions corresponding to 52.3, 34.1 and 10.4% of the surface area, respectively.

We recently described the structures of aryl­palladium iodide complexes also incidentally isolated from Sonogashira reaction products where the reactions were performed with a slight excess of the aryl iodide (Bosch, 2025 ▸). These results provide some insight into the fate of the palladium catalyst in the Sonogashira coupling reaction provided that one of the organic reagents is initially in slight excess.

## Synthesis and crystallization

The complex was isolated after a Sonogashira coupling reaction between 1,3,5-tri­fluoro-2-iodo­benzene and 2-ethynyl­pyrimidine. The reaction was performed with a slight excess, 1.05 molar equiv., of the ethynyl­pyrimidine. The product was detected as an orange crystalline impurity in the bulk product 2-(2,4,6-tri­fluoro­phenyl­ethyn­yl)pyrimidine isolated after flash chromatography with mixtures of hexane and ethyl acetate. Manual separation afforded small amounts of the complex in crystalline form suitable for single-crystal X-ray crystallography.

## Refinement

Crystal data, data collection and structure refinement details are summarized in Table 1 ▸.

## Supplementary Material

Crystal structure: contains datablock(s) I. DOI: 10.1107/S2414314625006364/vm4069sup1.cif

Structure factors: contains datablock(s) I. DOI: 10.1107/S2414314625006364/vm4069Isup2.hkl

Supporting information file. DOI: 10.1107/S2414314625006364/vm4069Isup3.cdx

CCDC reference: 2473412

Additional supporting information:  crystallographic information; 3D view; checkCIF report

## Figures and Tables

**Table 1:** Experimental details

Crystal data	
Chemical formula	[Pd(C_6_H_3_N_2_)_2_(C_18_H_15_P)_2_]
*M*_r_	837.15
Crystal system, space group	Monoclinic, *C*2/*c*
Temperature (K)	100
*a*, *b*, *c* (Å)	22.1529 (13), 13.7110 (13), 12.3853 (9)
β (°)	90.690 (2)
*V* (Å^3^)	3761.6 (5)
*Z*	4
Radiation type	Mo *K*α
μ (mm^−1^)	0.62
Crystal size (mm)	0.35 × 0.20 × 0.02

Data collection	
Diffractometer	Bruker APEXI CCD
Absorption correction	Multi-scan (*SADABS*; Krause *et al.*, 2015 ▸)
*T*_min_, *T*_max_	0.564, 0.746
No. of measured, independent and observed [*I* > 2σ(*I*)] reflections	24110, 4171, 3041
*R*_int_	0.079
(sin θ/λ)_max_ (Å^−1^)	0.642

Refinement	
*R*[*F*^2^ > 2σ(*F*^2^)], *wR*(*F*^2^), *S*	0.039, 0.091, 1.02
No. of reflections	4171
No. of parameters	250
H-atom treatment	H-atom parameters constrained
Δρ_max_, Δρ_min_ (e Å^−3^)	0.59, −0.66

Computer programs: *APEX2* and *SAINT* (Bruker, 2014 ▸), *SHELXT2018/2* (Sheldrick, 2015*a* ▸), *SHELXL2019/2* (Sheldrick, 2015*b* ▸) and *X-SEED-4* (Barbour, 2020 ▸).

## full crystallographic data

### (I) Crystal data

**Table:**

[Pd(C_6_H_3_N_2_)_2_(C_18_H_15_P)_2_]	*F*(000) = 1712
*M**_r_* = 837.15	*D*_x_ = 1.478 Mg m^−^^3^
Monoclinic, *C*2/*c*	Mo *K*α radiation, λ = 0.71073 Å
*a* = 22.1529 (13) Å	Cell parameters from 3308 reflections
*b* = 13.7110 (13) Å	θ = 2.4–23.5°
*c* = 12.3853 (9) Å	µ = 0.62 mm^−^^1^
β = 90.690 (2)°	*T* = 100 K
*V* = 3761.6 (5) Å^3^	Plate, orange
*Z* = 4	0.35 × 0.20 × 0.02 mm

### (I) Data collection

**Table:**

Bruker APEXI CCD diffractometer	3041 reflections with *I* > 2σ(*I*)
Detector resolution: 8.3660 pixels mm^-1^	*R*_int_ = 0.079
φ and ω scans	θ_max_ = 27.1°, θ_min_ = 1.8°
Absorption correction: multi-scan (SADABS; Krause *et al.*, 2015)	*h* = −28→28
*T*_min_ = 0.564, *T*_max_ = 0.746	*k* = −17→17
24110 measured reflections	*l* = −15→15
4171 independent reflections	

### (I) Refinement

**Table:**

Refinement on *F*^2^	Primary atom site location: dual
Least-squares matrix: full	Hydrogen site location: inferred from neighbouring sites
*R*[*F*^2^ > 2σ(*F*^2^)] = 0.039	H-atom parameters constrained
*wR*(*F*^2^) = 0.091	*w* = 1/[σ^2^(*F*_o_^2^) + (0.034*P*)^2^ + 7.3694*P*] where *P* = (*F*_o_^2^ + 2*F*_c_^2^)/3
*S* = 1.02	(Δ/σ)_max_ < 0.001
4171 reflections	Δρ_max_ = 0.59 e Å^−^^3^
250 parameters	Δρ_min_ = −0.66 e Å^−^^3^
0 restraints	

### (I) Special details

**Table:**

Geometry. All esds (except the esd in the dihedral angle between two l.s. planes) are estimated using the full covariance matrix. The cell esds are taken into account individually in the estimation of esds in distances, angles and torsion angles; correlations between esds in cell parameters are only used when they are defined by crystal symmetry. An approximate (isotropic) treatment of cell esds is used for estimating esds involving l.s. planes.

### (I) Fractional atomic coordinates and isotropic or equivalent isotropic displacement parameters (Å^2^)

**Table:**

	*x*	*y*	*z*	*U*_iso_*/*U*_eq_	
Pd1	0.750000	0.750000	0.500000	0.01796 (10)	
P1	0.66798 (4)	0.66217 (6)	0.56457 (6)	0.01975 (18)	
N1	0.58722 (14)	0.9407 (2)	0.2463 (3)	0.0449 (9)	
C1	0.70247 (14)	0.8220 (2)	0.3862 (2)	0.0209 (7)	
N2	0.67884 (15)	0.9460 (2)	0.1481 (2)	0.0372 (8)	
C2	0.67628 (14)	0.8677 (2)	0.3173 (2)	0.0211 (7)	
C3	0.64549 (15)	0.9208 (2)	0.2336 (3)	0.0252 (7)	
C4	0.5602 (2)	0.9855 (3)	0.1602 (4)	0.0638 (15)	
H4	0.518332	1.000109	0.163862	0.077*	
C5	0.5901 (2)	1.0098 (3)	0.0706 (4)	0.0581 (14)	
H5	0.569826	1.038911	0.010696	0.070*	
C6	0.6500 (2)	0.9914 (3)	0.0683 (3)	0.0473 (11)	
H6	0.672319	1.011791	0.007286	0.057*	
C7	0.59797 (15)	0.6599 (3)	0.4845 (2)	0.0253 (7)	
C8	0.56959 (17)	0.5749 (3)	0.4499 (3)	0.0372 (9)	
H8	0.587809	0.513304	0.463157	0.045*	
C9	0.51444 (19)	0.5807 (4)	0.3958 (3)	0.0513 (12)	
H9	0.495113	0.522482	0.372235	0.062*	
C10	0.48739 (17)	0.6689 (4)	0.3759 (3)	0.0456 (11)	
H10	0.449183	0.671727	0.340330	0.055*	
C11	0.51582 (16)	0.7524 (4)	0.4074 (3)	0.0444 (10)	
H11	0.497736	0.813819	0.392644	0.053*	
C12	0.57103 (15)	0.7480 (3)	0.4611 (3)	0.0351 (8)	
H12	0.590602	0.806847	0.482071	0.042*	
C13	0.68175 (14)	0.5330 (2)	0.5896 (2)	0.0213 (7)	
C14	0.67023 (15)	0.4879 (2)	0.6875 (3)	0.0252 (7)	
H14	0.658594	0.525676	0.748053	0.030*	
C15	0.67578 (15)	0.3873 (3)	0.6965 (3)	0.0304 (8)	
H15	0.668344	0.356419	0.763764	0.036*	
C16	0.69201 (16)	0.3318 (3)	0.6085 (3)	0.0338 (8)	
H16	0.694506	0.262872	0.614631	0.041*	
C17	0.70460 (17)	0.3766 (3)	0.5118 (3)	0.0344 (9)	
H17	0.716307	0.338504	0.451546	0.041*	
C18	0.70025 (16)	0.4772 (3)	0.5021 (3)	0.0306 (8)	
H18	0.709901	0.508013	0.435808	0.037*	
C19	0.64234 (14)	0.7144 (2)	0.6911 (3)	0.0216 (7)	
C20	0.58997 (16)	0.6818 (3)	0.7404 (3)	0.0329 (8)	
H20	0.567433	0.630066	0.708617	0.039*	
C21	0.57048 (17)	0.7238 (3)	0.8348 (3)	0.0387 (10)	
H21	0.535143	0.700015	0.868562	0.046*	
C22	0.60193 (17)	0.8000 (3)	0.8802 (3)	0.0372 (9)	
H22	0.588364	0.828918	0.945278	0.045*	
C23	0.65326 (16)	0.8344 (3)	0.8313 (3)	0.0309 (8)	
H23	0.674524	0.888152	0.861690	0.037*	
C24	0.67402 (15)	0.7907 (2)	0.7374 (3)	0.0256 (7)	
H24	0.710074	0.813407	0.705116	0.031*	

### (I) Atomic displacement parameters (Å^2^)

**Table:**

	*U*^11^	*U*^22^	*U*^33^	*U*^12^	*U*^13^	*U*^23^
Pd1	0.01900 (17)	0.01846 (16)	0.01646 (16)	−0.00124 (16)	0.00149 (12)	0.00134 (15)
P1	0.0215 (4)	0.0208 (4)	0.0170 (4)	−0.0020 (3)	0.0017 (3)	−0.0003 (3)
N1	0.0306 (18)	0.041 (2)	0.062 (2)	−0.0024 (15)	−0.0102 (16)	0.0225 (17)
C1	0.0194 (16)	0.0203 (16)	0.0232 (16)	−0.0026 (13)	0.0048 (13)	−0.0031 (13)
N2	0.058 (2)	0.0295 (17)	0.0238 (16)	0.0020 (15)	0.0020 (15)	0.0012 (13)
C2	0.0240 (17)	0.0203 (16)	0.0192 (16)	−0.0004 (13)	0.0023 (13)	−0.0015 (13)
C3	0.0289 (19)	0.0192 (17)	0.0275 (18)	−0.0030 (14)	−0.0054 (15)	0.0005 (14)
C4	0.036 (3)	0.059 (3)	0.096 (4)	−0.007 (2)	−0.025 (3)	0.032 (3)
C5	0.075 (4)	0.040 (3)	0.059 (3)	−0.015 (2)	−0.034 (3)	0.023 (2)
C6	0.080 (4)	0.032 (2)	0.029 (2)	−0.005 (2)	−0.010 (2)	0.0017 (17)
C7	0.0255 (18)	0.0334 (19)	0.0172 (16)	−0.0029 (15)	0.0028 (13)	0.0026 (14)
C8	0.040 (2)	0.040 (2)	0.031 (2)	−0.0081 (18)	−0.0111 (17)	0.0007 (17)
C9	0.043 (3)	0.067 (3)	0.043 (2)	−0.023 (2)	−0.016 (2)	0.002 (2)
C10	0.024 (2)	0.083 (3)	0.029 (2)	−0.006 (2)	−0.0055 (16)	0.014 (2)
C11	0.0243 (18)	0.064 (3)	0.045 (2)	0.006 (2)	0.0011 (16)	0.018 (2)
C12	0.0238 (17)	0.0376 (19)	0.044 (2)	0.0008 (19)	0.0023 (15)	0.003 (2)
C13	0.0206 (16)	0.0220 (16)	0.0213 (16)	−0.0035 (13)	−0.0001 (13)	0.0007 (13)
C14	0.0284 (19)	0.0279 (18)	0.0192 (16)	−0.0017 (15)	0.0011 (14)	0.0012 (14)
C15	0.0291 (19)	0.031 (2)	0.0306 (19)	−0.0011 (16)	0.0002 (15)	0.0108 (16)
C16	0.033 (2)	0.0231 (18)	0.046 (2)	−0.0005 (15)	0.0032 (17)	0.0059 (17)
C17	0.043 (2)	0.0261 (19)	0.034 (2)	0.0026 (17)	0.0046 (17)	−0.0057 (16)
C18	0.039 (2)	0.0305 (19)	0.0221 (17)	−0.0024 (16)	0.0057 (15)	0.0009 (15)
C19	0.0204 (16)	0.0213 (15)	0.0230 (16)	0.0003 (13)	0.0018 (13)	−0.0026 (13)
C20	0.031 (2)	0.040 (2)	0.0282 (19)	−0.0098 (17)	0.0043 (15)	−0.0097 (16)
C21	0.030 (2)	0.049 (3)	0.038 (2)	−0.0066 (17)	0.0128 (17)	−0.0106 (17)
C22	0.034 (2)	0.046 (2)	0.031 (2)	0.0018 (18)	0.0061 (17)	−0.0159 (18)
C23	0.030 (2)	0.034 (2)	0.0289 (19)	−0.0009 (16)	−0.0002 (15)	−0.0095 (16)
C24	0.0235 (18)	0.0278 (17)	0.0254 (17)	0.0002 (14)	0.0017 (14)	−0.0020 (14)

### (I) Geometric parameters (Å, º)

**Table:**

Pd1—C1	2.008 (3)	C8—C9	1.389 (5)
Pd1—C1^i^	2.008 (3)	C9—C10	1.371 (6)
Pd1—P1^i^	2.3293 (8)	C10—C11	1.362 (6)
Pd1—P1	2.3294 (8)	C11—C12	1.386 (5)
P1—C19	1.820 (3)	C13—C14	1.387 (4)
P1—C13	1.823 (3)	C13—C18	1.392 (4)
P1—C7	1.831 (3)	C14—C15	1.390 (5)
N1—C3	1.331 (5)	C15—C16	1.380 (5)
N1—C4	1.362 (5)	C16—C17	1.378 (5)
C1—C2	1.202 (4)	C17—C18	1.388 (5)
N2—C6	1.325 (5)	C19—C24	1.381 (4)
N2—C3	1.344 (4)	C19—C20	1.391 (5)
C2—C3	1.433 (4)	C20—C21	1.378 (5)
C4—C5	1.342 (6)	C21—C22	1.372 (5)
C5—C6	1.352 (6)	C22—C23	1.379 (5)
C7—C12	1.377 (5)	C23—C24	1.391 (5)
C7—C8	1.389 (5)		
			
C1—Pd1—C1^i^	180.0	C12—C7—P1	117.5 (3)
C1—Pd1—P1^i^	84.81 (9)	C8—C7—P1	124.0 (3)
C1^i^—Pd1—P1^i^	95.19 (9)	C9—C8—C7	119.6 (4)
C1—Pd1—P1	95.19 (9)	C10—C9—C8	121.1 (4)
C1^i^—Pd1—P1	84.81 (9)	C11—C10—C9	119.3 (4)
P1^i^—Pd1—P1	180.0	C10—C11—C12	120.3 (4)
C19—P1—C13	106.82 (14)	C7—C12—C11	121.1 (4)
C19—P1—C7	101.63 (15)	C14—C13—C18	119.6 (3)
C13—P1—C7	102.39 (15)	C14—C13—P1	123.3 (2)
C19—P1—Pd1	110.17 (11)	C18—C13—P1	116.9 (2)
C13—P1—Pd1	115.53 (11)	C13—C14—C15	119.8 (3)
C7—P1—Pd1	118.82 (11)	C16—C15—C14	120.4 (3)
C3—N1—C4	114.7 (4)	C17—C16—C15	119.9 (3)
C2—C1—Pd1	177.0 (3)	C16—C17—C18	120.3 (3)
C6—N2—C3	116.4 (4)	C17—C18—C13	120.0 (3)
C1—C2—C3	178.9 (4)	C24—C19—C20	118.9 (3)
N1—C3—N2	125.7 (3)	C24—C19—P1	119.6 (2)
N1—C3—C2	118.2 (3)	C20—C19—P1	121.4 (2)
N2—C3—C2	116.1 (3)	C21—C20—C19	120.6 (3)
C5—C4—N1	122.8 (4)	C22—C21—C20	120.2 (3)
C4—C5—C6	117.7 (4)	C21—C22—C23	119.8 (3)
N2—C6—C5	122.5 (4)	C22—C23—C24	120.2 (3)
C12—C7—C8	118.5 (3)	C19—C24—C23	120.1 (3)

Symmetry code: (i) −*x*+3/2, −*y*+3/2, −*z*+1.
